# *lepidium-like*, a Naturally Occurring Mutant of *Capsella bursa-pastoris*, and Its Implications on the Evolution of Petal Loss in Cruciferae

**DOI:** 10.3389/fpls.2021.714711

**Published:** 2021-11-25

**Authors:** Anna V. Klepikova, Elina D. Shnayder, Artem S. Kasianov, Margarita V. Remizowa, Dmitry D. Sokoloff, Aleksey A. Penin

**Affiliations:** ^1^Institute for Information Transmission Problems of the Russian Academy of Sciences, Moscow, Russia; ^2^Faculty of Biology, Lomonosov Moscow State University, Moscow, Russia

**Keywords:** apetaly, *Capsella bursa-pastoris*, flower development, genetic analysis, heterochrony, morphological evolution, polyploidy, Cruciferae

## Abstract

Naturally occurring mutants whose phenotype recapitulates the changes that distinguish closely related species are of special interest from the evolutionary point of view. They can give a key about the genetic control of the changes that led to speciation. In this study, we described *lepidium-like* (*lel*), a naturally occurring variety of an allotetraploid species *Capsella bursa-pastoris* that is characterized by the typical loss of all four petals. In some cases, one or two basal flowers in the raceme had one or two small petals. The number and structure of other floral organs are not affected. Our study of flower development in the mutant showed that once initiated, petals either cease further development and cannot be traced in anthetic flowers or sometimes develop to various degrees. *lel* plants showed an earlier beginning of floral organ initiation and delayed petal initiation compared to the wild-type plants. *lel* phenotype has a wide geographical distribution, being found at the northern extremity of the species range as well as in the central part. The genetic analysis of inheritance demonstrated that *lel* phenotype is controlled by two independent loci. While the flower in the family Cruciferae generally has a very stable structure (i.e., four sepals, four petals, six stamens, and two carpels), several deviations from this ground plan are known, in particular in the genus *Lepidium*, *C. bursa-pastoris* is an emerging model for the study of polyploidy (which is also very widespread in Cruciferae); the identification and characterization of the apetalous mutant lays a foundation for further research of morphological evolution in polyploids.

## Introduction

An important feature of many angiosperm flowers is the occurrence of structures serving for the attraction of pollinators and protection of reproductive organs. Even though the views on homologies and evolutionary origin of angiosperm petals remain controversial (e.g., Ronse De Craene, 2007), the appearance of a double perianth with pronounced differences between calyx and corolla is a key innovation of eudicots. In contrast, sepals and petals (as well as nectaries) do not directly influence plant viability and fertility, and consequently, these organs are in certain sense optional in floral morphology (Irish, 2008). A loss of petals is widespread across eudicots. Apetaly can emerge as a homeotic transformation of petals into other organs such as stamens, petal loss, or petal suppression. These phenomena can affect all or only some petals of a flower. In several eudicot families, the recurrent loss of petals considerably increases the variation of flower ground plan (Endress, 2006; Pieper et al., 2016). Apart from wind-pollinated lineages, apetaly is especially common among selfing plants since they do not require pollinators (Culley and Klooster, 2007; Sharples et al., 2021).

Among eudicots, the family Cruciferae (Brassicaceae) demonstrates a particularly stable floral structure. The vast majority of species possess flowers with four sepals, four petals, six stamens, and two carpels, thus making the family an attractive model for studies of flower development and evolution (Tucker, 2000; Endress, 2006). At the same time, some species from various genera of Cruciferae display a reduction of corolla providing a remarkable example of parallel evolution. The genetic control of apetaly has been studied in *Lepidium* L., where petal losses took place many times in course of evolution and characterized some taxonomically recognized species (Endress, 1992; Bowman et al., 1999; Lee et al., 2002). Petal reduction in *Lepidium* is usual, although not always (*Lepidium alashanicum* H. L. Yang) accompanied by a reduction in stamen number (Bowman and Smyth, 1998; Bowman et al., 1999; Zhou et al., 2001), which makes comparisons of flower development in different species complicated, because more than one parameter should be compared. Detailed developmental data are available for the Australian species of *Lepidium* with different flower ground plans (Bowman and Smyth, 1998), and the early stages of the development were covered for dioecious species *Lepidium sisymbrioides* Hook. f. and its closest relatives *Lepidium naufragorum* Garn.-Jones & D. A. Norton and *Lepidium tenuicaule* Kirk (Soza et al., 2014). The genetic analysis of *Lepidium* is significantly hampered by the difficulty of interspecies crosses: species with contrasting phenotypes failed to hybridize, although other crosses have even resulted in F_3_ hybrids (Lee et al., 2002). Among other Cruciferae, naturally occurring apetalous forms can be found in *Capsella* Medik., *Cardamine* L., *Microlepidium* F. Muell., *Rorippa* Scop., *Subularia* L., and *Thellungiella* E.O. Schulz (Opiz, 1821; Murbeck, 1918; Zhou et al., 2001; Appel and Al-Shehbaz, 2003; Kim et al., 2010; Pieper et al., 2016). In the agricultural species *Brassica napus* L., petal loss in certain cultivars increases productivity (Rao et al., 1991). The polygenic genetic control of apetaly (along with environmental influence) has been identified in *Cardamine hirsuta* L. (Pieper et al., 2016).

*Capsella bursa-pastoris* (L.) Medik. is a widespread recent allotetraploid and a model in the studies of polyploidy (Douglas et al., 2015; Kasianov et al., 2017). Similar to its parental species, *Capsella orientalis* Klokov and *Capsella rubella* Reut., *C. bursa-pastoris* is a self-compatible plant (Slotte et al., 2012; Sicard et al., 2016). In the 19th century, apetalous *C. bursa-pastoris* was discovered in Europe and considered as a distinct species *Capsella apetala* Opiz (1821). Current taxonomic accounts place *C. apetala* in the synonymy of *C. bursa-pastoris* (Nutt et al., 2006). The apetaly of plants described as *C. apetala* was caused by the homeotic transformation of petals into stamens making the flower “decandric” (with 10 stamens, Hintz et al., 2006). This condition clearly differs from the common pattern found in many eudicots where 10 stamens form two whorls of 5 (5 + 5). When 10 stamens are present in the *Capsella* mutant, their arrangement can be described as 4 + 2 + 4. The mutant was termed *Stamenoid petals* (*Spe*) and is supposedly caused by a mutation in the regulatory sequence of *Capsella AGAMOUS* (Nutt et al., 2006; Ziermann et al., 2009; Hameister et al., 2013).

We discovered plants of *C. bursa-pastoris* with all flowers within an individual either lacking any petals or having less than four petals in Moscow, Russia. Unlike *Spe*, the variation that we called *lepidium-like* (*lel*) is characterized only by decreased petal number without a homeotic transformation. We aimed to investigate the geographical distribution of the *lel* variation along with its morphological and genetic analysis. We found *lel C. bursa-pastoris* to be widespread in the North-West of European Russia and other parts of Europe. It also occurs in Siberia, Japan, and the United States. We determined the mode of inheritance of apetaly and mapped the regions of the localization of the affected genes.

## Materials and Methods

### Plant Growth

Seeds of *C. bursa-pastoris* were stratified on Kvitko medium (Kvitko, 1960) at 4°C for 7–14 days. Seeds were grown in a climate chamber (Pol-Eko Aparatura, Poland) under long-day (16-h light/8-h dark cycle) conditions at 22°C. After the appearance of the first leaf, plants were transferred to 1:3 vermiculite:soil and were grown at 21–23°C in a growing chamber.

### Floral Phenotype Analysis

Wild-type [i.e., wild-type moscow-1 (wt-msc-1)] and apetalous mutant [i.e., *lepidium-like-moscow-1* (*lel-msc-1*)] varieties of natural origin were grown in a growing chamber in conditions that prevented outcrossing for four generations. A maternal *lel-msc-1* plant was emasculated and crossed with a paternal wt-msc-1 plant. For all six F_1_ plants, petal number was counted for all flowers on the main and secondary inflorescence axes without flower removal.

For the analysis of the inheritance mode, F_1_ plants were self-pollinated and the floral phenotype of 93 F_2_ progenies was analyzed as described earlier. If a plant did not have secondary axes, the shoot tip was resected to remove the apical dominance.

### Statistical Analysis

The chi-square (χ^2^) test was used to assess the differences between the theoretically expected ratio and the observed ratio of phenotypes in F_2_ progeny. χ^2^ test was applied using chisq.test function from R package “Stats” (R Core Team, 2021), with a significance threshold (*p*) of 0.05.

### Scanning Electron Microscopy

Flower structure and development of wt-msc-1 and *lel-msc-1* plants were studied using scanning electron microscopy (SEM). The material was fixed and stored in 70% ethanol and dissected in 96% ethanol. Parts of young inflorescences and flowers were dehydrated in 96% ethanol followed by a mixture of 96% ethanol and 100% acetone (1:1) and three changes of 100% acetone. The dehydrated material was critical-point-dried using a Hitachi HCP-2 (Tokyo, Japan) critical point dryer, coated with gold and palladium using an Eiko IB-3 ion-coater (Tokyo, Japan) and observed using a CamScan S-2 (Cambridge Instruments, London, United Kingdom) and a JSM-6380LA SEM (JEOL, Tokyo, Japan) at 20 kV, all at the Moscow State University.

### DNA Extraction, Library Preparation, and Sequencing

The DNA of 46 (23 wild-type and 23 *lel*) plants was extracted from frozen at –20°C leaves using the cetrimonium bromide (CTAB) method (Doyle and Doyle, 1987). Notably, 65 ng of DNA of each plant was taken and mixed, forming wild-type and mutant pools separately. Sequencing libraries were prepared using TruSeq DNA Sample Preparation Kits (Illumina, San Diego, CA, United States), according to the instructions of the manufacturer. Libraries were sequenced on Illumina HiSeq 2000 with 100-bp paired reads.

### Sequenced Read Preparation and Mapping

Reads were mapped on *C. bursa-pastoris* reference genome (Kasianov et al., 2017; *Capsella bursa-pastoris* Database, 2020) using CLC Genomics Workbench 20.0.3 (CLC Bio, Denmark) with the following settings: “Match score = 1; Mismatch cost = 3; Cost of insertions and deletions = Linear gap cost; Insertion cost = 3; Deletion cost = 3; Length fraction = 1.0; Similarity fraction = 0.94; Global alignment = No; Non-specific match handling = Ignore.”

### Single Nucleotide Polymorphism-Calling of *lel* Parent

The single nucleotide polymorphisms (SNPs) were called using CLC Genomics Workbench 9.5.4 (CLC Bio, Denmark) with following settings: “Ploidy = 2; Ignore positions with coverage above = 250; Ignore broken pairs = Yes; Ignore Non-specific matches = Reads; Minimum coverage = 5; Minimum count = 5; Minimum frequency (%) = 95.0; Base quality filter = Yes; Neighborhood radius = 5; Minimum central quality = 20; Minimum neighborhood quality = 15; Read direction filter = Yes; Direction frequency (%) = 5.0; Relative read direction filter = Yes; Significance (%) = 1.0; Read position filter = Yes; Significance (%) = 1.0.”

### Single Nucleotide Polymorphism-Calling for Wild-Type and Mutant Pools

The SNPs were called using CLC Genomics Workbench 9.5.4 (CLC Bio, Denmark) with the following settings: “Ploidy = 2; Ignore positions with coverage above = 100; Restrict calling to target regions = Not set; Ignore broken pairs = Yes; Ignore non-specific matches = Reads; Minimum coverage = 2; Minimum count = 2; Minimum frequency (%) = 15.0; Base quality filter = Yes; Neighborhood radius = 5; Minimum central quality = 20; Minimum neighborhood quality = 15; Read direction filter = Yes; Direction frequency (%) = 5.0; Relative read direction filter = Yes; Significance (%) = 1.0; Read position filter = Yes; Significance (%) = 1.0 s.”

### Single Nucleotide Polymorphism Index Calculation

For SNP Index counting, MutMap version 2.3.2 software was used. The alignments for mutant pool and wild-type pool were used as input parameters for MutMap.

### Data Availability Statement

The original contributions presented in this study are publicly available. These data can be found here as follows: The genome sequences of *lel* parent, wild-type F_2_ pool, and mutant F_2_ pool are available in the NCBI Sequence Read Archive (project ID PRJNA655599).

## Results

### Natural Apetalous Mutant of *Capsella bursa-pastoris*

**FIGURE 1:**
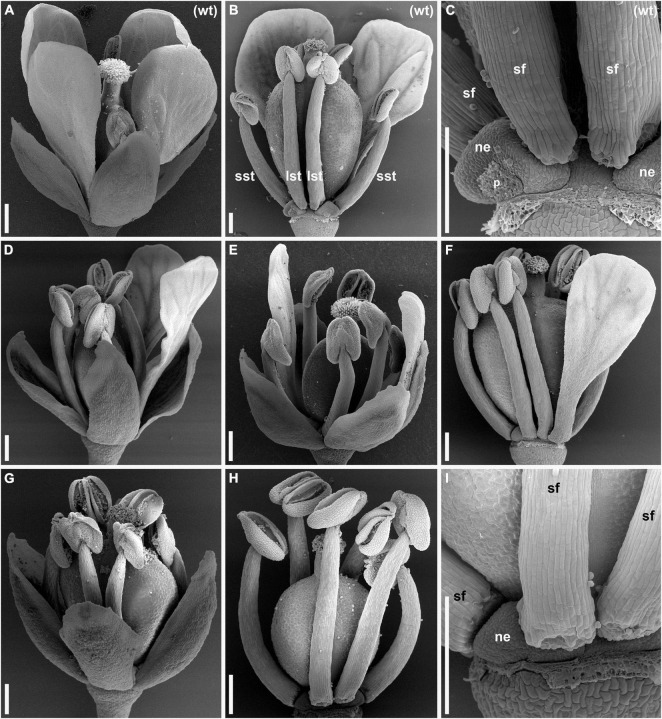
Anthetic flowers of wild type **(A–C)** and *lepidium-like* (*lel*) **(D–I)** plants of *Capsella bursa-pastoris* [scanning electron microscopy (SEM)]. **(A)** Side view of wild-type flower with four conspicuous petals. **(B)** Wild-type flower with all sepals and two of four petals removed. **(C)** Detail of **(B)** showing a nectary that is closely associated with a petal. **(D,E)** Flowers with two petals. **(D)** The two petals are in adjacent positions. **(E)** The two petals are in diagonal positions. **(F)** Flower with one petal; sepals are removed. **(G–I)** Flowers having no petals. **(G)** General view. **(H)** Flower with sepals removed. **(I)** Detail of **(H)** showing a nectary. lst, long stamen; ne, nectary; p, scar of removed petal; sf, stamen filament; sst, short stamen; wt, wild type. Scale bars: 300 μm.

We discovered a novel natural mutant of *C. bursa-pastoris* in Moscow, Russia, and named it *lel* since its flowers resemble those of some species of the genus *Lepidium* in the typical absence of visible petals. We extracted mutant and wild-type plants from the same localities and established several accessions. Accessions used in all subsequent experiments in this study were named wt-msc-1 variety and *lel-msc-1* (apetalous plants). Both wt-msc-1 and *lel-msc-1* were propagated by single seed descent for four generations in conditions that prevented outcrossing and did not show any segregation in all four generations. Thus, *lel-msc-1*, as well as wt-msc-1, had stably inherited phenotypes. Flowers of the accession wt-msc-1 (Figures 1A,B) had a typical Cruciferae flower morphology with four sepals, four petals, six stamens, and two carpels throughout the inflorescence with marginal variation in the uppermost part of the inflorescence where a few three-petaled flowers emerged. Almost all anthetic flowers of the accession *lel-msc-1* had no visible petals (Figures 1G,H). In some cases, one or two basal flowers per raceme had one or two small petals recognizable without magnification (Figures 1D–F). As soon as flowers of the mutant either have no visible petals or have less than four petals, below for brevity, we call the mutant apetalous. Other aspects of flower structure, such as the occurrence of four sepals, six stamens, two carpels, and four nectaries, were the same in the wild type and the mutant (Figure 1).

### Flower Development

**FIGURE 2:**
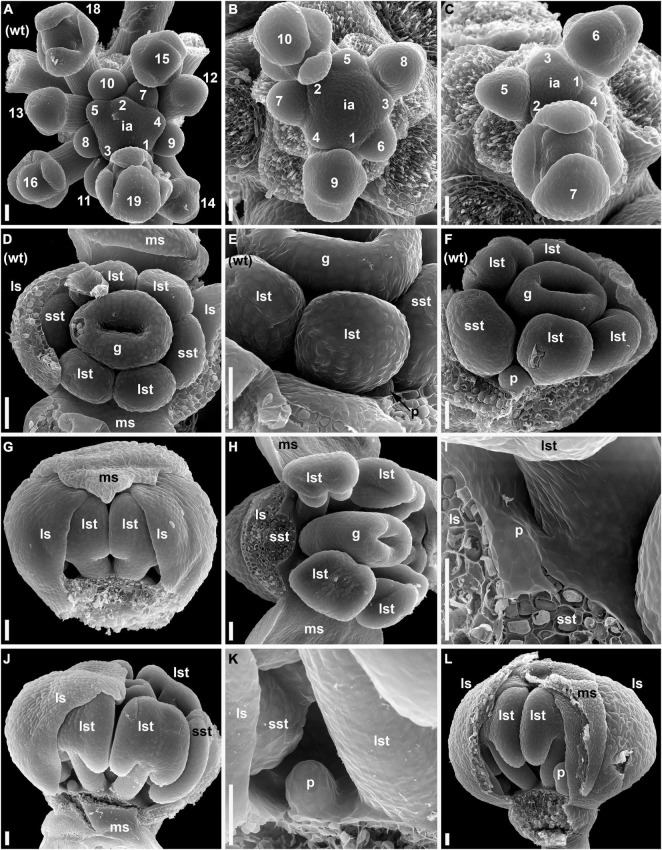
Flower development in the wild-type **(A,D–F)** and *lel* plants **(B,C,G–L)** of *C. bursa-pastoris* (SEM). **(A–C)** Inflorescence apices with flowers at the sequential stages of early development. The flowers are numbered sequentially from the youngest to the oldest. **(D–F)** The earliest stages of petal development in the wild type. **(D,E)** A flower with lateral sepals removed. **(D)** Top view. **(E)** Detail of side view. It is noted that the very small petal and the virtual absence of stamen filaments. **(F)** A flower with sepals removed. The petal is larger than in **(E)**, and very short stamen filaments are present. **(G–L)** Early petal development in *lel* plants. **(G)** Side view of a flower with one median sepal removed. It is noted that the gaps on either side of the visible pair of long stamens. A small petal primordium is present in the right-hand gap. **(H)** A flower with a sepal and a stamen removed. **(I)** Detail of **(H)** showing a petal primordium. **(J)** Side view of a flower with three of four sepals removed. A young petal is present left to the removed median sepal. There is no petal right to the median sepal (although there is enough space for its initiation). **(K)** Detail of **(J)** showing the petal. **(L)** Side view of flower with a median sepal partially removed. There is a petal right to the median sepal, but no petal left to it. g, gynoecium; ia, inflorescence apex; ls, lateral sepal; lst, long stamens; ms, median sepal; p, petal; sst, short stamens; wt, wild type. Scale bars: 30 μm.

Flowers of the wild-type and *lel* plants have the same general sequence of floral organ initiation. Sepals are the first organs to be initiated and are followed by stamens and then carpels. Petals, when initiated, are the last organs to appear in the flower. A detailed comparative description of flower development in the wild-type and *lel* plants is provided in Supplementary Material 1. In this study, we highlighted the observed developmental differences between the two accessions. In the developing inflorescences of the wild type, at least 10 youngest flowers closest to the inflorescence apex yet have no evidence of organ initiation (Figure 2A). When the flower commences to organ initiation, a well-developed pedicel can be recognized (flowers 12 and 13 in Figure 2A). In *lel* plants, the first evidence of organ initiation takes place earlier than in the wild type, at least when the time is measured in plastochrons of the inflorescence axis (5–8 plastochrons from the apex, Figures 2B,C). The pedicel is short or inconspicuous at the beginning of organ initiation (Figures 2B,C). In *lel* plants, the median adaxial sepal appears to be more retarded in development than in the wild type. It may be speculated that due to the close contact to the inflorescence axis, the median adaxial sepal of *lel* has no enough space to develop without pressure, which causes retardation (e.g., Figure 2C, flower 6). Alternatively, a physiological influence of the inflorescence apex may play a role here. The floral apex is triangular in outline right before sepal initiation in *lel* plants (Figure 2C, flower 5), which may indicate the earliest manifestation of the median abaxial and lateral sepals. In the development of the wild type, the petals can be first recorded at the stage when the young anthers yet appear almost sessile (Figures 2D–F). In *lel* plants, the first evidence of petal initiation can be traced only after the appearance of clearly visible stamen filaments (Figures 2G–I). The petals do not initiate in all four corners of the flower (Figures 2J–L). Unequal petal size can be noted at early and later developmental stages (Supplementary Material 1).

### Geographical Distribution of Apetalous *Capsella*

**FIGURE 3:**
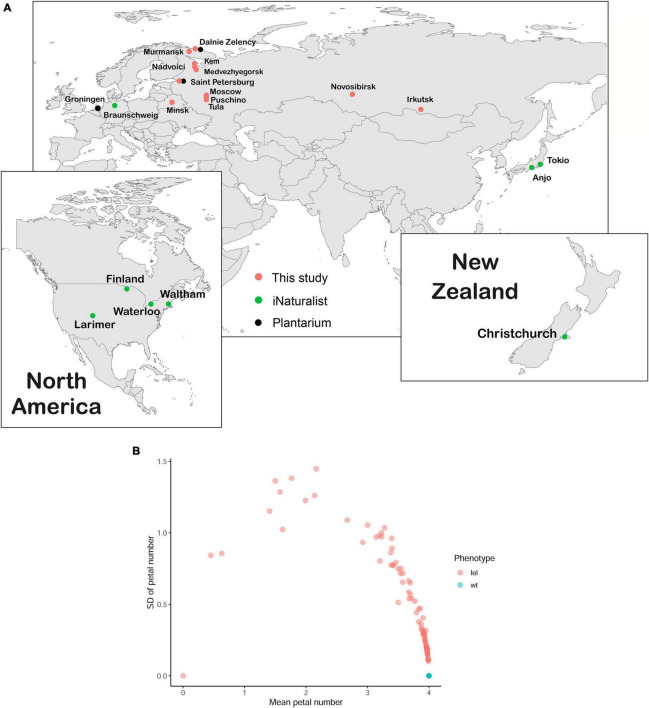
Geographical distribution and phenotype variance of apetalous *C. bursa-pastoris*. **(A)** The geographical distribution of *lel* variety, mapped using database exploring (Plantarium and iNaturalist) and field search. Each dot represents a single location where apetalous *C. bursa-pastoris* was found. **(B)** The dependence of SD of petal number on average petal number. For each plant, petals were counted in each flower on the main and secondary inflorescence axes, and then, average and SD were calculated for every plant. Each dot represents one plant; it is noted that all wild-type plants overlap.

*Capsella bursa-pastoris* has a worldwide distribution. We assessed the range of the apetalous *C. bursa-pastoris* using both online and field research. We addressed the largest online herbaria with queries “*Capsella apetala*” and “*Capsella bursa-pastoris var. apetala*” with a result of 18 samples from 4 herbaria (Supplementary Table 1). The analysis of images in public databases Plantarium (180 photographs, Plantarium Database, 2017) and iNaturalist (>6,000 images, iNaturalist Database, 2019) resulted in 3 and 10 images of apetalous *Capsella*, respectively (Supplementary Table 1). According to the Plantarium database, the distribution of the apetalous form covered Saint-Petersburg and Dalniye Zelentsy (North-West of Russia) and Groningen (Netherlands) (Figure 3A, black circles). In the iNaturalist database, the apetalous *Capsella* is recorded from the United States, New Zealand, Japan, and Germany (Figure 3A, green circles).

During field studies, we found apetalous *C. bursa-pastoris* phenotypically similar to *lel-msc-1* in Saint-Petersburg, Puschino, and Tula (European Russia, Figure 3A, pink circles) and in Minsk (Belarus, Figure 3A, pink circles). The extensive field search in North-West Russia (Republic of Karelia and Murmansk region) allowed to map the geographical distribution of the apetalous phenotype: plants with more or less pronounced apetaly were found in Nadvoitsy, Kem, Medvezhyegorsk, Murmansk, and Dalniye Zelentsy (Figure 3A, pink circles).

### Phenotype of F_1_ Hybrids and Phenotypic Ratio in F_2_ Progenies

Quantitative traits such as the number and size of organs often have a polygenic control (Irish, 2008). In Cruciferae, the loss of petals can be caused by a single gene mutation, as in *Arabidopsis thaliana* (Lampugnani et al., 2013), or be associated with multiple quantitative trait loci (QTLs), as was determined for *C. hirsuta* and *B. napus* (Pieper et al., 2016; Yu et al., 2016). To analyze the mode of the inheritance of the apetalous phenotype, we performed a cross between wt-msc-1 and *lel-msc-1* lines, using *lel-msc-1* as a maternal plant. The F_1_ hybrid plants had an intermediate phenotype: up to 10 basal flowers on the inflorescence axis had 4 petals as the wild type, then the number of petals decreased along the length of the inflorescence, and more apical flowers had either three or four petals. The intermediate degree of apetaly in F_1_ plants is the evidence of a codominant inheritance. F_1_ hybrids were self-pollinated to yield F_2_ seeds.

We analyzed the number of petals in each flower in 103 plants that began to flower out of 120 seeds sowed (Supplementary Tables 2, 3). The floral phenotypes formed a wide spectrum from completely apetalous to wild-type plants (Figure 3B). Notably, 19 plants did not show any petal loss either on the main inflorescence axis or on the secondary axes. For 84 plants, the mean number of petals in flowers varied between 0.00 and 3.98 (Figure 3B; Supplementary Figure 1). Only three plants had a strong apetalous phenotype (Figure 3B; Supplementary Tables 2, 3) similar to that of *lel* plants, which had 0.48 petals on average with SD = 0.22. In the case of codominance monogenic inheritance of a trait, we would have observed three phenotypic classes with segregation 1:2:1. For 103 plants that would have corresponded to 25.75 (1/4 wt phenotype): 51.50 (1/2 intermediate phenotype): 25.75 (1/4 *lel* phenotype) plants, we actually observed 19 plants with wild-type phenotype, 80 plants with intermediate phenotype, and 3 plants with *lel* phenotype. Based on the statistical analysis (i.e., the χ^2^ test), we rejected the hypothesis on monogenic inheritance (χ^2^ = 38.767, df = 2, *p*-value = 3.818e-09). Based on the same test, the observed segregation fits the hypothesis on digenic inheritance, which implies theoretical segregation 19.3125 (3/16 wt phenotype): 83.6875 (13/16 mutant phenotype) (χ^2^ = 0.006, df = 1, *p*-value = 0.937, which means that null hypothesis cannot be rejected). Thus, based on the ratio of wild-type/mutant plants, we inferred a digenic inheritance of apetaly. As soon as the wild-type phenotypic class corresponds to 3/16, we hypothesized that the mutation in one gene is codominant while in the other – recessive. Otherwise, if both mutations had been recessive, we would not have observed intermediate phenotype in the F_1_ generation, and the frequency of wild-type phenotype in F_2_ would have been 9/16, and if both mutations had been codominant, the frequency of wild-type phenotype in F_2_ would have been 1/16.

### Trait-Linked Regions in *Capsella bursa-pastoris* Genome

**FIGURE 4:**
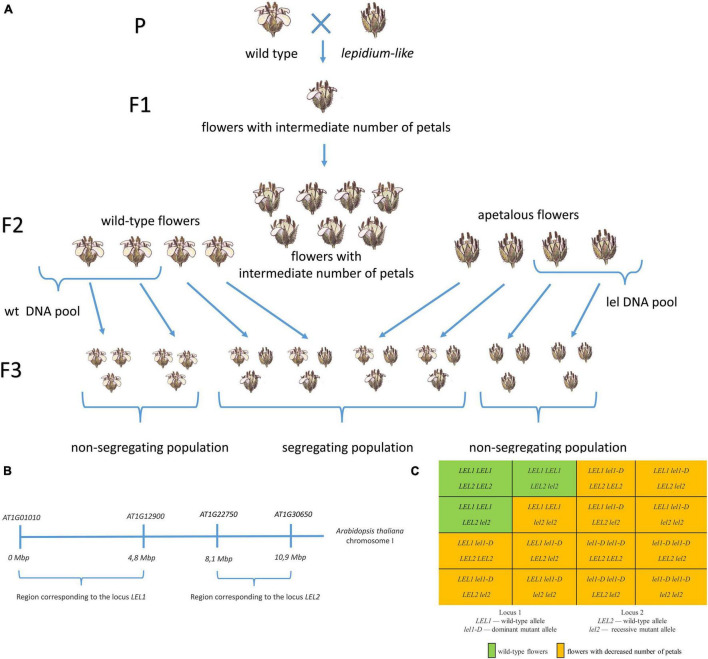
The SNP-based identification of loci associated with apetaly in the *C. bursa-pastoris* genome. **(A)** The workflow of F_2_ pool construction for DNA sequencing. Wild-type and *lel* plants were crossed to obtain F_1_ progeny with intermediate phenotype. The self-cross of F_1_ plants resulted in segregating the F_2_ population. We selected F_2_ plants with wild-type or severe apetalous phenotype and analyzed their F_3_ progeny from self-pollination. Leaves of F_2_ plants of which F_3_ progeny did not show phenotype segregation were used for DNA extraction and sequencing. **(B)** The region of *Arabidopsis thaliana* chromosome I with corresponding apetaly-associated loci 1 and 2. **(C)** The Punnett square for F_2_ progeny of wild-type and *lel C. bursa-pastoris*.

We aimed to identify genome regions associated with apetalous phenotype using SNP-based gene mapping. First, we collected pools of wild-type and mutant progeny (Figure 4A). For this purpose, we analyzed the floral phenotype of 426 F_2_ plants along with the harvesting and freezing of leaves (Supplementary Tables 4, 5). We selected F_2_ plants with the wild-type and almost wild-type phenotypes (i.e., four petals in each flower, one flower with three petals was allowed, 120 plants total) or a severe mutant phenotype (with a mean number of petals less than 1.5, 31 plants). Then, we analyzed F_3_ progeny from self-pollination (20 seeds of F_3_ for each F_2_). F_2_ plants whose progeny showed only parental phenotype were selected for the subsequent analysis (23 wild-type and 23 *lel*). DNA was extracted from leaves and pooled in equal quantity, and two pools (i.e., wild-type and mutant) were subjected to Illumina whole-genome sequencing.

We created a database of SNPs between the wild-type and mutant parental lines. As the genome of the wt-msc-1 line was sequenced in our previous study (Kasianov et al., 2017), we sequenced a parental mutant (*lel-msc-1*) plant. After the quality control, the *lel-msc-1* reads were mapped on the reference genome of *Capsella*, and SNP-calling was performed to find the polymorphisms that differentiate wild-type and mutant genomes (refer to “Materials and Methods” section). We obtained 217,099 high-confidence SNPs between parental lines.

We mapped the reads of the wild-type and *lel* pools on the *C. bursa-pastoris* genome and performed SNP-calling. The obtained SNPs were filtered against a database of parental variants. For each scaffold, the mean frequency of *lel* SNPs was counted for the wild-type and mutant pools. We extracted scaffolds based on the following criteria: maximal scaffold average frequency of *lel* SNPs in a mutant pool and minimal scaffold average frequency of *lel* SNPs in the wild-type pool (Supplementary Figure 2).

The segregation of apetalous phenotype in the F_2_ generation suggested a digenic inheritance of petal loss. As expected, we found two regions associated with apetaly: the first region was located in subgenome A and the second one in subgenome B (Figure 4B). The search for associated regions was repeated with MutMap software (version 2.3.2, Abe et al., 2012), and the resulting contigs were the same (Supplementary Figure 3).

Subgenome A contains five scaffolds matching the abovementioned criteria (hereinafter locus 1). The scaffolds were ≈3.3 Mb in total and contained 749 annotated genes. We localized the region of the *A. thaliana* genome corresponding to the selected scaffolds based on the presence of orthologous genes. All scaffolds correspond to one region of the *A. thaliana* chromosome I (genes *AT1G01010*–*AT1G12900*, Figure 4B). These scaffolds were characterized by ≈0.7 frequency of *lel* parental SNPs and by ≈0.05 frequency of wt parental SNPs in a mutant pool, which implies the presence of frequency ratio of SNP 4 *lel* parental:2 wt parental. Thus, we observed a dominant inheritance of the first gene controlling apetaly as the mutant pool consists of 1 part *lel1-D lel1-D* and 2 part *LEL1 lel1-D* (ratio 4 *lel1-D*:*2 LEL1*).

Eight scaffolds passing our criteria belonged to subgenome B (hereinafter locus 2). The total length of the scaffolds was ≈2.5 Mb, and 525 genes were annotated in them. All scaffolds match a single region of the *A. thaliana* chromosome I (genes *AT1G22750*–*AT1G30650*, Figure 4B). These scaffolds had almost 1.0 frequency of *lel* parental SPNs in a mutant pool (≈0.97) and by ≈0.10 frequency of wt parental SNPs, hence, only the *lel2 lel2* genotype results in an apetalous phenotype. Therefore, we suggested the recessive inheritance of the second gene identified.

Notably, the types of inheritance are different for the two loci involved: dominant for the locus from the subgenome A and recessive for the locus from the subgenome B. However, both mutations lead to the same phenotype that implies the opposite functions of these loci.

Taken together, we identified the genotypes of wild-type plants as *LEL1 LEL1 LEL2 LEL2* or *LEL1 LEL1 LEL2 lel2*, which form 3/13 of the F_2_ population, and other genotypes result in a wide variety of the apetaly. This result fits the phenotype ratio of F_2_ plants (Figure 4C) and intermediate apetaly of F_1_ hybrids.

We found 1,278 SNPs in 320 genes from scaffolds associated with locus 1, from which 5 resulted in premature stop codons. The dominant inheritance of the locus 1 causative gene can be explained by changes in its promoter structure rather than in the gene body. The intergenic regions of locus 1 harbored 1,220 SNPs. Locus 2 had 572 SNPs in 265 genes with 4 stops. As we have identified the recessive mode of inheritance of apetaly in locus 2, we analyzed *A. thaliana* orthologs of four *C. bursa-pastoris* genes harboring premature stop codons: genes *AT1G29230* (*CBL-INTERACTING PROTEIN KINASE 18*) encoding a member of the SNF1-related kinase gene family, *AT1G23210* (*GLYCOSYL HYDROLASE 9B6*), *AT1G26410* (*ATBBE6*) where the product belonged to the flavin adenine dinucleotide (FAD)-binding Berberine family, and *AT1G26640* (*ISOPENTENYL PHOSPHATE KINASE*) encoding an isopentenyl phosphate kinase that regulates terpenoid compounds. The role of these genes in the development of *A. thaliana* is not yet established, and the expression pattern of genes in the *A. thaliana* transcriptome map (Klepikova et al., 2016; Transcriptome Variation Analysis Database, 2020) does not lead to a suggestion of any of these genes as a candidate because none of them has a predominant expression pattern in petals or developing flowers.

The dominance of locus 1 together with the significant number of affected genes complicates a direct search for candidate genes, so we used *A. thaliana* orthologs to identify candidate genes. Locus 1 covered 1,374 *A. thaliana* genes from which 552 had orthologs in subgenome A of *C. bursa-pastoris*. Of note, 835 genes were placed in trait-associated locus 2 in *A. thaliana* with 374 orthologs among *C. bursa-pastoris* genes. We did not find any strong candidates for either *LEL1* or *LEL2* (e.g., *STERILE APETALA*, *APETALA3*, *PISTILLATA*, and *PETAL LOSS*), which suggests a divergent genetic control of petal development between *A. thaliana* and *C. bursa-pastoris* despite their close phylogenetic relationships.

## Discussion

The wild-type and *lel* plants of *C. bursa-pastoris* differ not only in definitive morphology but also in sequence and timing of organ initiation. The *lel* phenotype starts calyx initiation earlier and petal initiation later than the wild-type plants. Notably, the petals are the last organs to be initiated in both phenotypes. The flowers of most Cruciferae have a stable ground plan (reviewed in Endress, 1992; Ronse De Craene, 2010; Ronse De Craene and Brockington, 2013), but the visible sequence of organ initiation varies considerably among examined members of the family (Erbar and Leins, 1997). The differences appear to be taxon-specific and cover relative timing of initiation of petals, i.e., long and short stamens. Some Cruciferae such as *Iberis sempervirens* L. and *Isatis tinctoria* L. are found to have fully acropetal patterns of organ initiation, such as calyx - > corolla - > short stamens - > long stamens - > gynoecium. Other representatives show various deviations from the acropetal pattern (Erbar and Leins, 1997; Leins and Erbar, 2010). *Cochlearia officinalis* L. and *C. bursa-pastoris* were reported as taxa with petals initiated after the appearance of all stamens and before gynoecium initiation, but no illustration was provided for *Capsella* (Leins and Erbar, 2010). Our data support early light microscopic observations (Coulter and Chamberlain, 1903) that petal initiation takes place even later, such as after gynoecium initiation in the wild-type and the *lel* mutant of *Capsella*, so that the sequence is calyx - > short and long stamens - > gynoecium - > corolla. The analysis of our data along with the images available in the literature (Polowick and Sawhney, 1986; Smyth et al., 1990; Erbar and Leins, 1997; Bowman and Smyth, 1998) clearly shows that the petal initiation even in wild-type *C. bursa-pastoris* takes place at a significantly later developmental stage than in other examined Cruciferae, such as *C. officinalis*. The differences between wt and *lel* are in the relative timing of initiation, number, size, and morphological differentiation of the petals. In *lel*, the petals are initiated considerably later than in the wt, and their number is fewer than four. Also, the petals of *lel* flowers, in the rare cases when they reach a considerable size at anthesis, are often less differentiated compared to wt, sometimes with only a claw developed (Supplementary Material 1). The (partial) loss of petals in the *lel* morphotype can be observed as a further continuation of the general tendency of delayed petal initiation that characterizes this species.

An important shared feature of *lel* plants of *Capsella* (this study) and apetalous species of *Lepidium* (Bowman and Smyth, 1998) is that petal primordia do initiate during flower development, though the petals remain very small and not recognizable in anthetic flowers. At least some petals were initiated in all examined flowers of *lel* plants of *Capsella.* However, petal initiation is not that delayed relative to gynoecium development in *Lepidium* as it does in *Capsella*. The four small petal primordia arise at the same time as the stamen primordia in examined *Lepidium* species (Bowman and Smyth, 1998). Interestingly, the developmental pattern of androecium reduction differs from that of corolla reduction in *Lepidium*: species with less than six stamens do not develop primordia of missing stamens (Bowman and Smyth, 1998). Thus, there is a suppression of petals but complete loss of some stamens in *Lepidium* spp.

The family Cruciferae belongs to rosids, a clade whose members show a developmental tendency to petal retardation. In many taxa with a double perianth, the organs are initiated acropetally with petals delayed till very late developmental stages (Endress, 2011). Some species demonstrate late initiation of petals, which ultimately results in the non-acropetal patterns of organ inception (reviewed by Sattler, 1973; Rudall, 2010; Remizowa, 2019). The petals are completely lost in some genera of Rosaceae and Fabaceae. Moreover, among predominantly wind-pollinated taxa, some entire orders or families such as Fagales, Urticaceae, Moraceae, or Eleagnaceae lack petals (Endress, 2010). Apparently, the loss of corolla is governed by different mechanisms in these cases but might have evolved for similar reasons at least in some cases. Apetalous *C. bursa-pastoris*, such as the *lel* variety described in our study, is a promising system to clarify the genetic processes underlying morphological evolution.

Our study highlights the limitations of the candidate gene approach and shows that the emergence of similar phenotypes in related species (known as Vavilov’s homologous series) is not necessarily based on the action of orthologous loci (as it is usually thought, e.g., Folta, 2015). While there are many cases where the candidate gene approach allowed to find the genetic basis of a trait in a non-model species (Kramer and Hodges, 2010; Wickland and Hanzawa, 2015; Martínez-Gómez et al., 2021), this may reflect the publication bias rather than the actual frequency of the involvement of orthologous genes. The direct identification of loci for the traits of interest, without the reliance on candidate genes, is much more time-consuming and requires developed genomic resources for non-model species. Recent advances in DNA sequencing and accompanying technologies are already enabling this (Al-Mssallem et al., 2013; Zhu et al., 2019), and we expect that this approach will provide new discoveries in the next few years.

## Data Availability Statement

The original contributions presented in the study are publicly available. This data can be found here: Genome sequences of lel parent, wild-type F2 pool and mutant F2 pool are available in the NCBI Sequence Read Archive (project ID PRJNA655599).

## Author Contributions

AKl analyzed F_2_ phenotypes for pooled DNA sequencing, collected plant material, and wrote the manuscript draft. ES analyzed F_2_ phenotypes for the inheritance inference and geographical distribution and generated images. AKa participated in bioinformatics analysis. MR analyzed SEM images and participated in manuscript writing. DS obtained the SEM images of flower development, analyzed SEM images, and participated in manuscript writing. AP coordinated the study, discovered the apetalous line, performed the genetic analysis, obtained the SEM images of anthetic flowers, participated in the bioinformatics analysis, designed the final figures, and participated in manuscript writing. All authors read and approved the final manuscript.

## Conflict of Interest

The authors declare that the research was conducted in the absence of any commercial or financial relationships that could be construed as a potential conflict of interest.

## Publisher’s Note

All claims expressed in this article are solely those of the authors and do not necessarily represent those of their affiliated organizations, or those of the publisher, the editors and the reviewers. Any product that may be evaluated in this article, or claim that may be made by its manufacturer, is not guaranteed or endorsed by the publisher.
