# Supplementary material for: lepidium-like, a Naturally Occurring Mutant of Capsella bursa-pastoris, and Its Implications on the Evolution of Petal Loss in Cruciferae
Source: Front Plant Sci. 2021 Nov 25;12:714711. doi: 10.3389/fpls.2021.714711 (PMC8656458; doi:10.3389/fpls.2021.714711)
Supplement: Supplementary file 1 [file Data_Sheet_1.zip › Supplementary Figures.PDF]

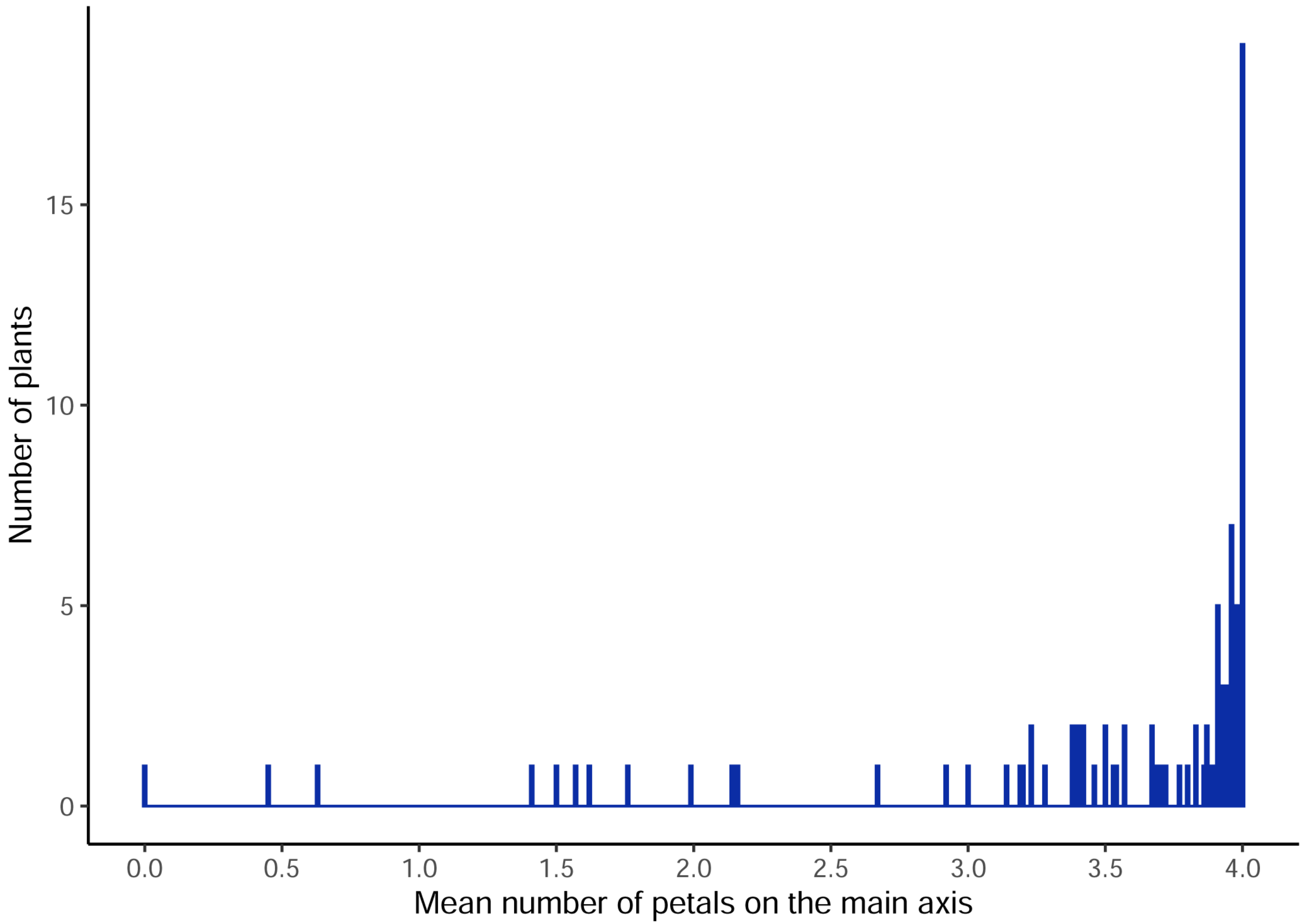

Supplementary Figure 1. The histogram of the mean number of petals of the plant.

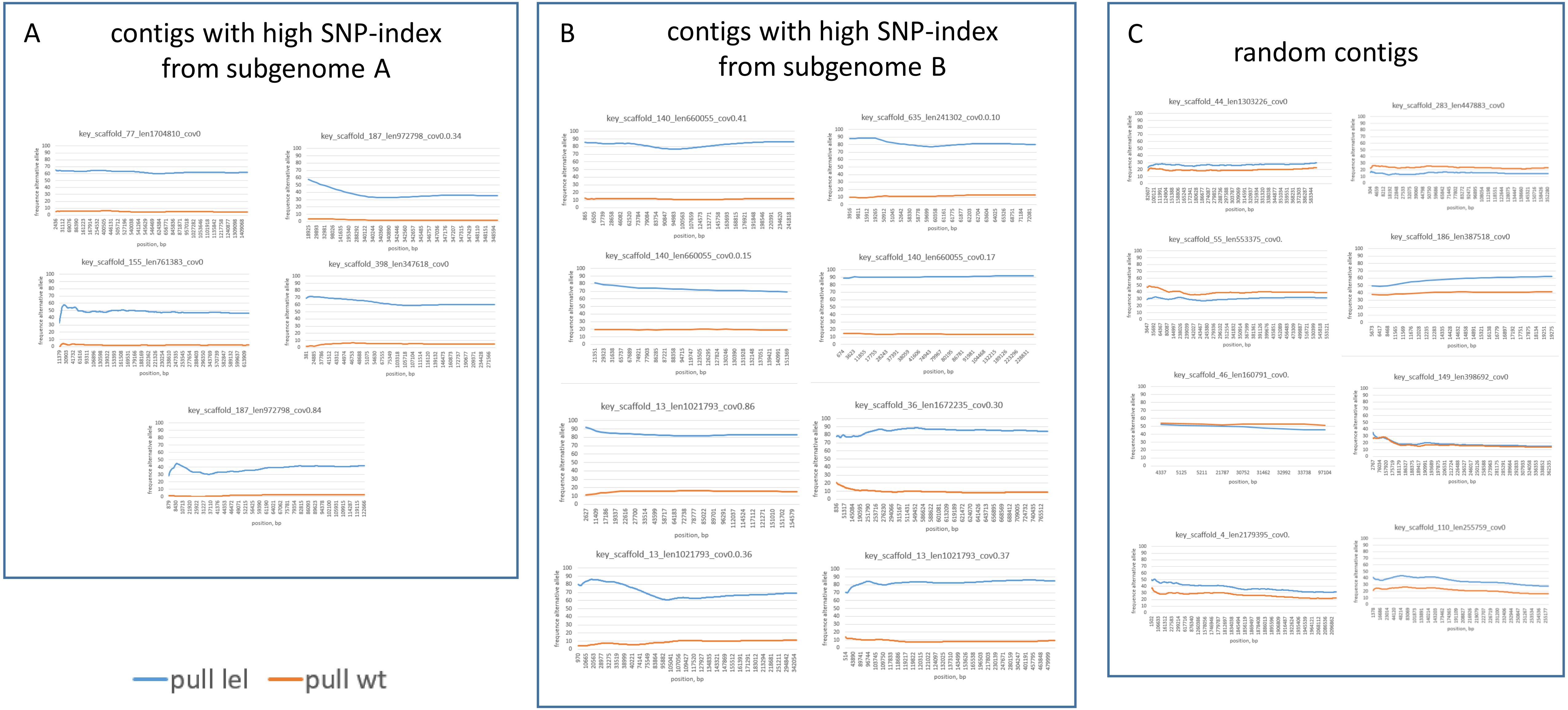

Supplementary Figure 2. Frequency of alternative SNP (wt - reference genome, lel - alternate) averaged over a sliding window. A. The contigs belonged to subgenome A with maximal average *lel* SNPs frequency in a mutant pool and minimal scaffold average *lel* SNPs frequency in the wild-type pool; B. The contigs belonged to subgenome B with maximal average *lel* SNPs frequency in a mutant pool and minimal scaffold average *lel* SNPs frequency in the wild-type pool; C. Randomly selected contigs.

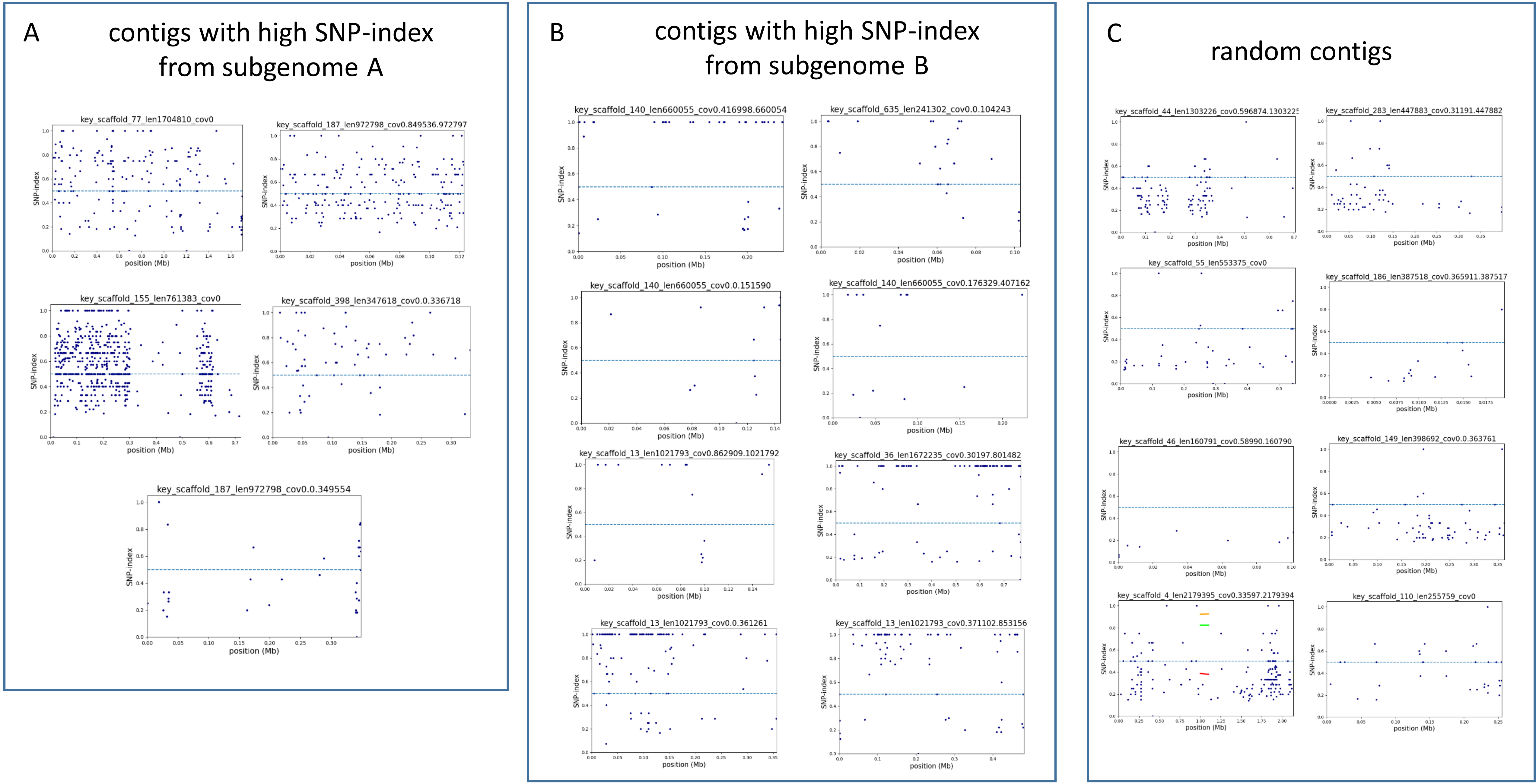

Supplementary Figure 3. The SNP index calculated by MutMap software for selected contigs. A. The contigs belonged to subgenome A with high SNP index; B. The contigs belonged to subgenome B with high SNP index; C. Randomly selected contigs.
