# Supplementary material for: lepidium-like, a Naturally Occurring Mutant of Capsella bursa-pastoris, and Its Implications on the Evolution of Petal Loss in Cruciferae
Source: Front Plant Sci. 2021 Nov 25;12:714711. doi: 10.3389/fpls.2021.714711 (PMC8656458; doi:10.3389/fpls.2021.714711)
Supplement: Supplementary file 1 [file Data_Sheet_1.zip › Supplementary Material 1.PDF]

Supplement 1.

Detailed description of flower development in wild type and *lel* plants of *Capsella bursa-pastoris*

In the wild type (Figure S1A-C) and *lel* plants (Figure S4A-C), flowers initiate acropetally on the inflorescence apex in a sequence that approaches a Fibonacci spiral. In the wild type, the first evidence of organ initiation in flowers can be traced at the distance of c. 11-13 plastochrons from the inflorescence apex. The flower primordium elongates considerably by this time, and when the flower commences to organ initiation a well-developed pedicel can be recognized (flower 10 in Figure S1B). In *lel* plants, the first evidence of organ initiation takes place earlier than in the wild type, at least when the time is measured in plastochrons of the inflorescence axis (5-8 plastochrons from the apex, Figure S4A-C). The pedicel is short or inconspicuous at the beginning of organ initiation (Figure S4B, C).

In both the wild type and *lel* plants, the first organs to be initiated are the sepals (Figures S1A-C, S4A-E). In *lel* plants (Figure S1B, flower 9; Figure S1C, flower 6), the median adaxial sepal appears to be more retarded in development than in the wild type. The floral apex is triangular in outline right before sepal initiation in *lel* plants (Figure S4C, flower 5), which may indicate the earliest manifestation of the median abaxial and lateral sepals.

The four sepals soon form an almost complete meristematic belt surrounding the flower (Figures S1A-D, S4E, F). The shape of this belt is complex and cannot be visualized in top views only, as the lateral sepals are situated at lower levels than the median sepals. At late developmental stages (when initiation of all organs is completed), the median abaxial sepal is much longer than the median adaxial sepal in the wild type (Figure S1A, flower 19, S1C, flower 23) as well as in *lel* plants (Figure S4B, flower 10). In the wild type, the difference in length between the median abaxial and median adaxial sepals is not that manifested at earlier stages (Figure S1D, G-I), and some images of early flowers even suggest that the adaxial sepal is slightly longer than the abaxial one (Figure S1E). In contrast, in *lel* plants, the median abaxial sepal is longer than the median adaxial one already at early stages (Figure S4B, F-I). The two lateral sepals appear shorter than the median ones throughout development (but note their lower insertion level) in both wild type (Figures S1, S2A) and *lel* plants (Figure S4).

After sepal initiation, the young flower bends inwards by more extensive pedicel and receptacle growth on the abaxial side. The next organs to be initiated are the stamens. The floral apex is hemispherical before stamen initiation (wild type: Figure S1A, flower 16, S1B, flower ; *lel*: Figure S4E, F). The two short stamens and two common primordia of long stamens are initiated simultaneously (wild type: Figure S1D; *lel*: Figure S4G-I). (Here and below, we refer to relative stamen length in anthetic flowers to distinguish the two types of stamens). Each of the two short stamens can be first recognized as a fold-like (i.e., very low and strongly transversally elongated) primordium in front of a lateral sepal. The four long stamens are initiated as two common primordia. Each of them ultimately produces two long stamens. A common primordium is formed in front of each median sepal. The two long-stamen common primordia are attached higher up on the flower axis than the short-stamen primordia. They are strongly elongated transversally, but more massive than the short-stamen primordia. Further growth of the long-stamen common primordia is localized at their lateral sides, so that two individual stamens can be recognized (Figure S1F), though a basal connection between the pairs of the long stamens can be traced up to early stages of gynoecium development (wild type: Figure S2A, C, D; *lel*: Figure S5A, B). Some images suggest a slight developmental retardation of the adaxial pair of long stamen primordia relative to the abaxial pair in *lel* plants (Figure S5A). The floral apex remaining after the androecium initiation is pronouncedly

elliptic in outline, with the longer axis in the transverse plane of the flower (wild type: Figures S1I, S2A-D; *lel*: Figures S4F,S5A). It soon becomes dome-shaped and can be recognized as the young gynoecium (wild type: Figure S2F, G; *lel*: Figure S5B). The carpels appear *ab initio* completely united, as a transversally elongated ringwall (wild type: Figure S2H, I; *lel*: Figure S5E, F). No petals can be seen yet at this stage in both wild type and *lel* plants. The six stamens are clearly distinct, but stamen filaments are yet absent.

The petals appear in the four corners of the flower in the radii between adjacent long and short stamens (Figures S3B, C, E-G, S5I, S6A-D). In the wild type, the youngest petals are recorded at the time of the first evidence of elongation of stamen filaments (Figure S2A-F). The petals of the wild type plants remain small until the latest stages of flower development (Figure S3H); ultimately they first elongate as strip-like structures (Figure S3I) and then expand laterally (Figure 1A-C). In *lel* plants, the first evidence of petal initiation can be traced only after the appearance of clearly visible stamen filaments (Figure S5H, I). The petals do not initiate in all four corners of the flower (Figure S6A, C, E, F). Unequal petal size can be seen at early (Figure S6D) and later (Figure S6G) developmental stages. In the wild type and *lel* plants, nectaries are the last structures to appear in course of flower development. There are four nectaries per flower. When petals are present, each nectary is associated with petal base (Fig. 1C, D).

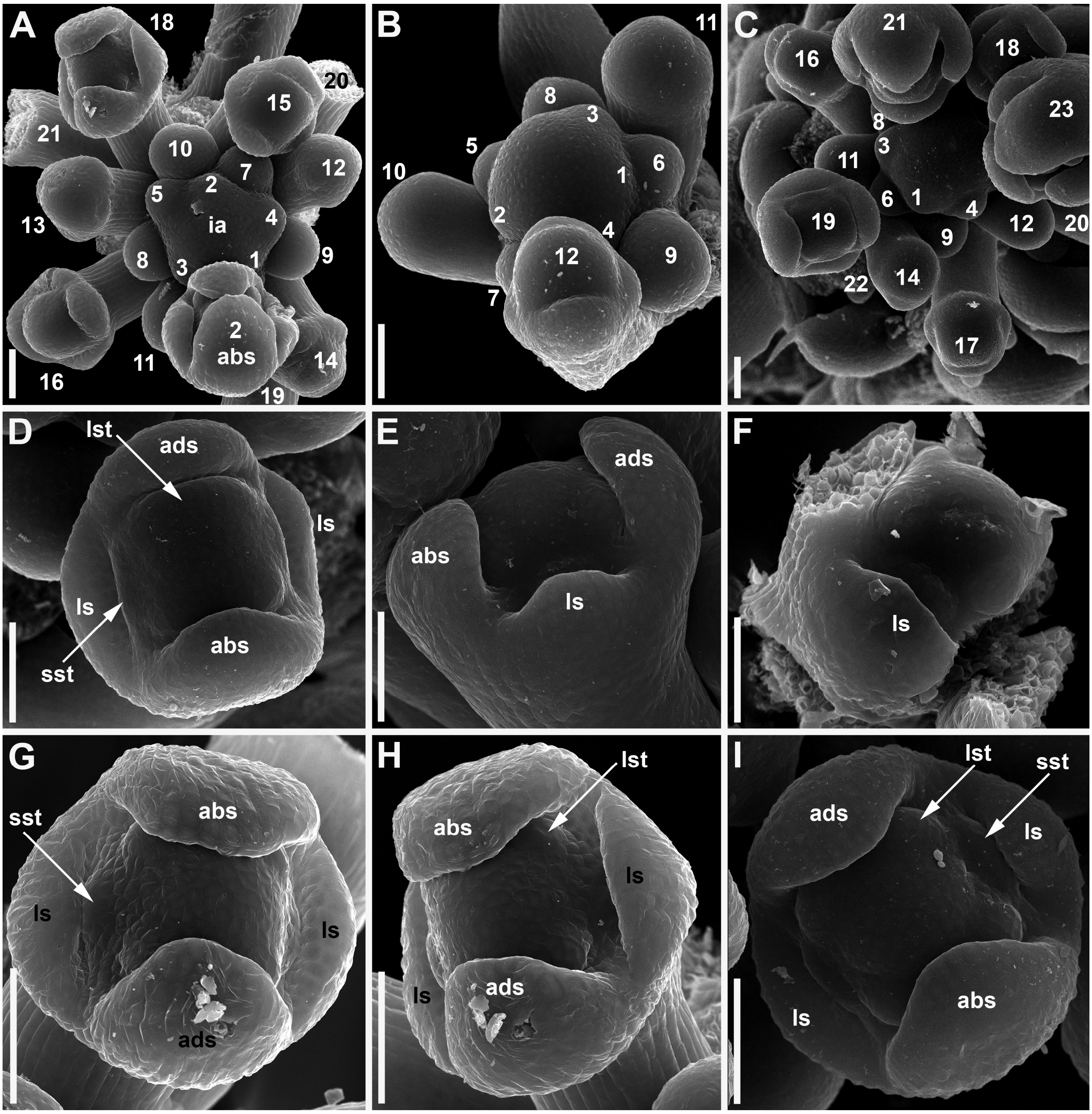

Figure S1. Initiation and early development of calyx and androecium in the wild type material of *C. bursa-pastoris* (SEM). (**A-C**). Inflorescence apices with flowers at sequential stages of early development. Flowers are numbered from the youngest to older ones. (**D**). Top view of flower with two short stamen primordia and two common primordia of long stamens. (**E**). Side view of stage similar to or slightly later than in (**D**). (**F**). Stage similar to that in (**D**), three sepals removed. (**G,H**). Different views of flower with calyx and androecium initiated. Note the absence of petals and carpels and almost equal size of the two median sepals that are both longer than in (**D**). (**I**). Similar to (**G,H**), but note a raised elliptic area between long stamen primordia that might be interpreted as the earliest evidence of gynoecium initiation. abs, abaxial sepal; ads, adaxial sepal; ia, inflorescence apex; ls, lateral sepal; lst, common primordium of long long stamens; sst, primordium of short stamens. Scale bars: 50 µm.

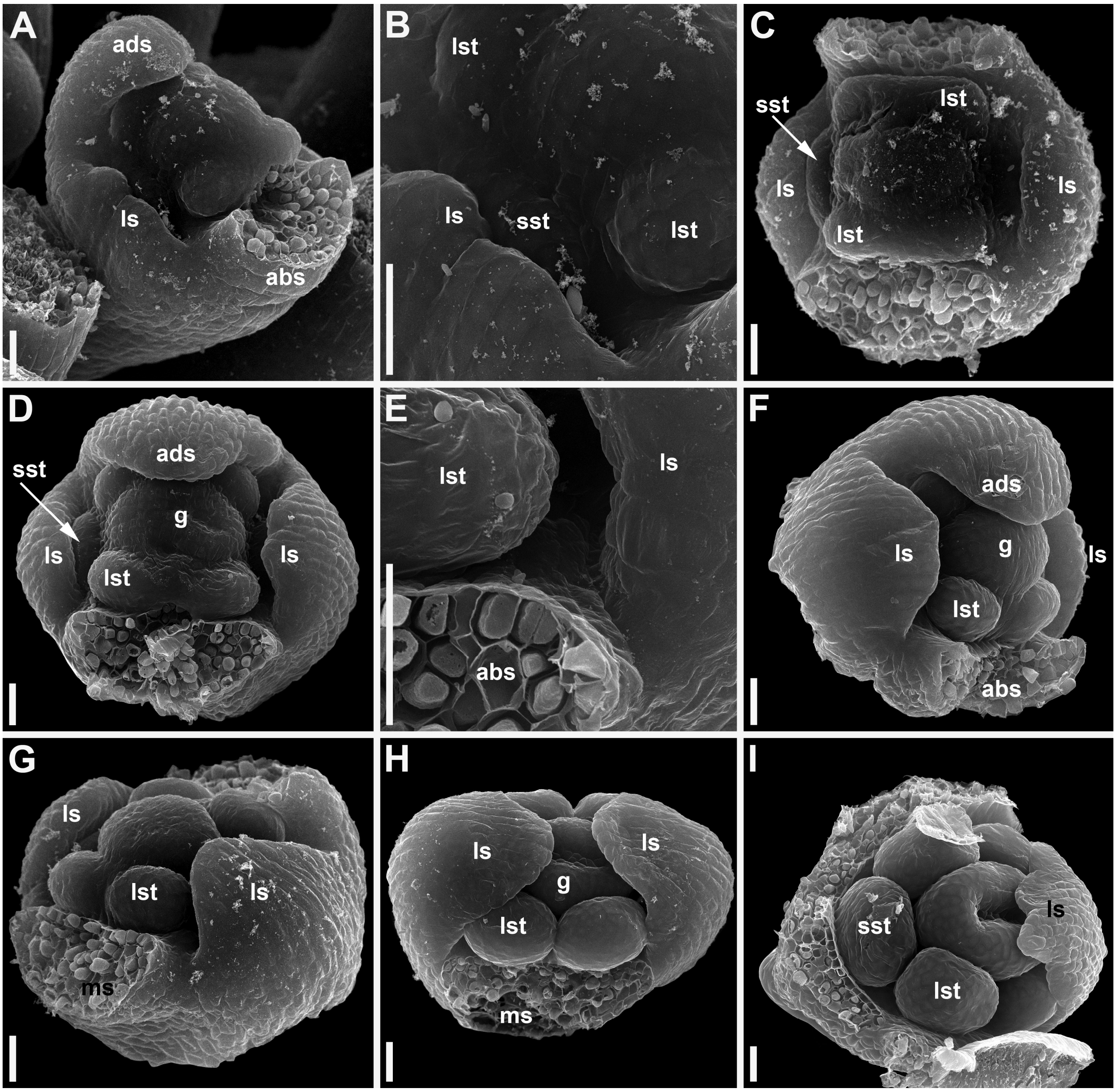

Figure S2. Early gynoecium development in wild type material of *C. bursa-pastoris* (SEM). Note the absence of petals. (**A**). Flower with median abaxial sepal removed (it was larger than the remaining median adaxial sepal). (**B**). Detail of (**A**) showing the absence of petals. (**C**). Flower with both median sepals removed. Note that the pairs of the long stamens are still undivided. Like in (**A**), the raised elliptic central area that might be interpreted as gynoecium can be seen. (**D**). Appearance of individual long stamens. (**E**). Detail of (**D**) showing the absence of petals. (**F,G**). Further growth of the raised central area. (**H,I**). Gynoecium ringwall is present, no traces of connection between the pairs of long stamens can be seen. abs, abaxial sepal; ads, adaxial sepal; g, gynoecium; ls, lateral sepal; lst, long stamens (i.e., those that will be long stamens in anthetic flowers); ms, median sepal; sst, short stamens (i.e, those that will be short stamens in anthetic flowers). Scale bars: 20 µm.

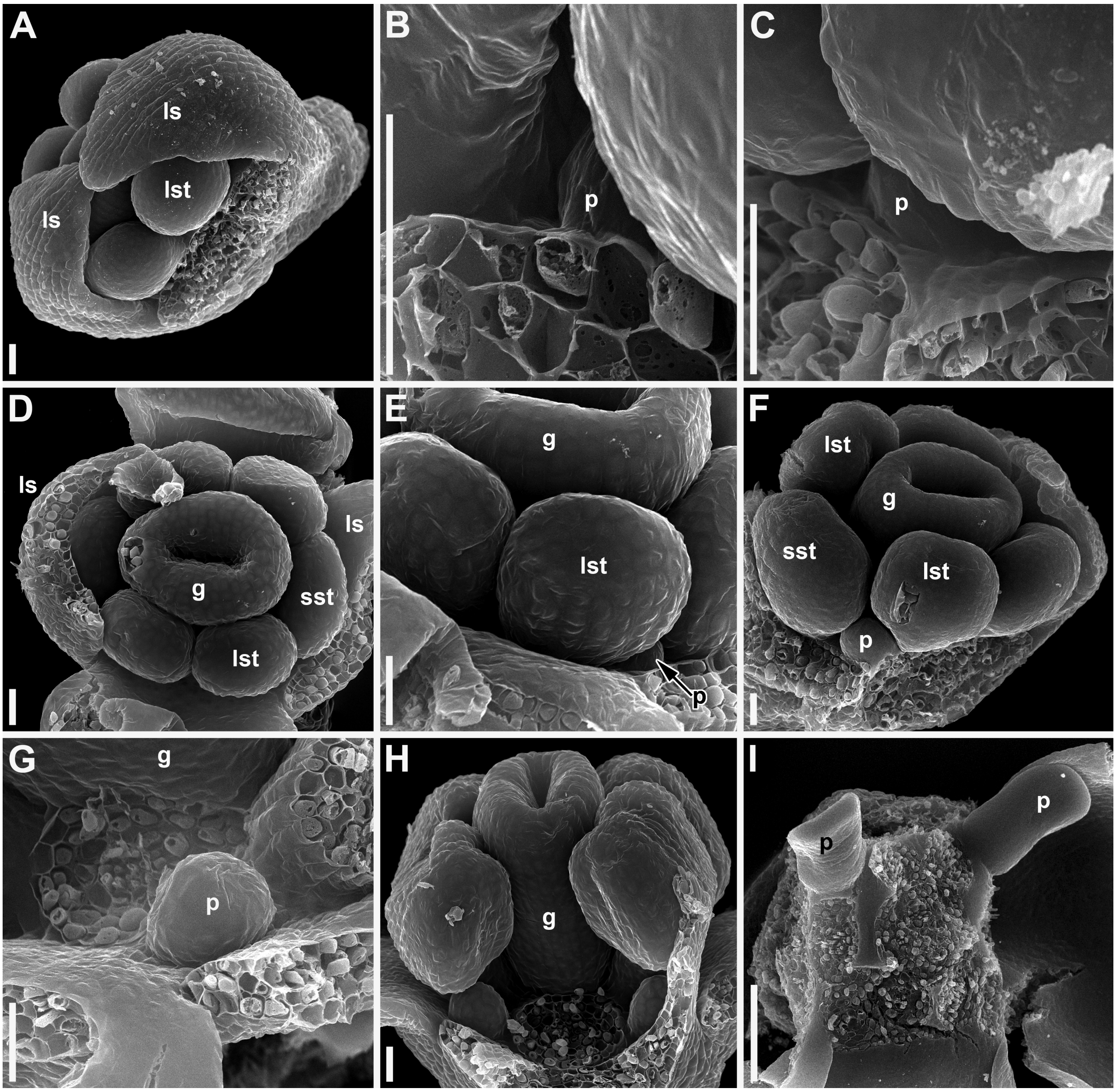

Figure S3. Corolla development in wild type material of *C. bursa-pastoris* (SEM). (**A,B**). Different views of flower at the earliest stage of corolla development. Both median sepals removed. (**A**). Top view. Petals are not visible, because they are hidden by stamens. (**B**). Detail of side view showing one of the four petals. (**C**). Similar to (**B**) in another flower. (**D,E**). Flower with lateral sepals removed. (**D**). Top view. (**E**). Detail of side view. Note the very small petal and the virtual absence of stamen filaments. (**F**). Flower with sepals removed. Petals larger than in (**E**), very short stamen filaments are present. (**G**). Stage similar to (**F**), sepals and stamens removed to show entire petal. (**H**). Calyx and a short stamen removed. Stamens much more differentiated than in (**F**), petals are still short. (**I**). Much later stage than in (**H**), all organs removed except two of four petals. abs, abaxial sepal; ads, adaxial sepal; g, gynoecium; ls, lateral sepal; lst, long stamens (i.e., those that will be long stamens in anthetic flowers); ms, median sepal; p, petal; sst, short stamens (i.e, those that will be short stamens in anthetic flowers). Scale bars: 20 µm (**A-H**), 100 µm (**I**).

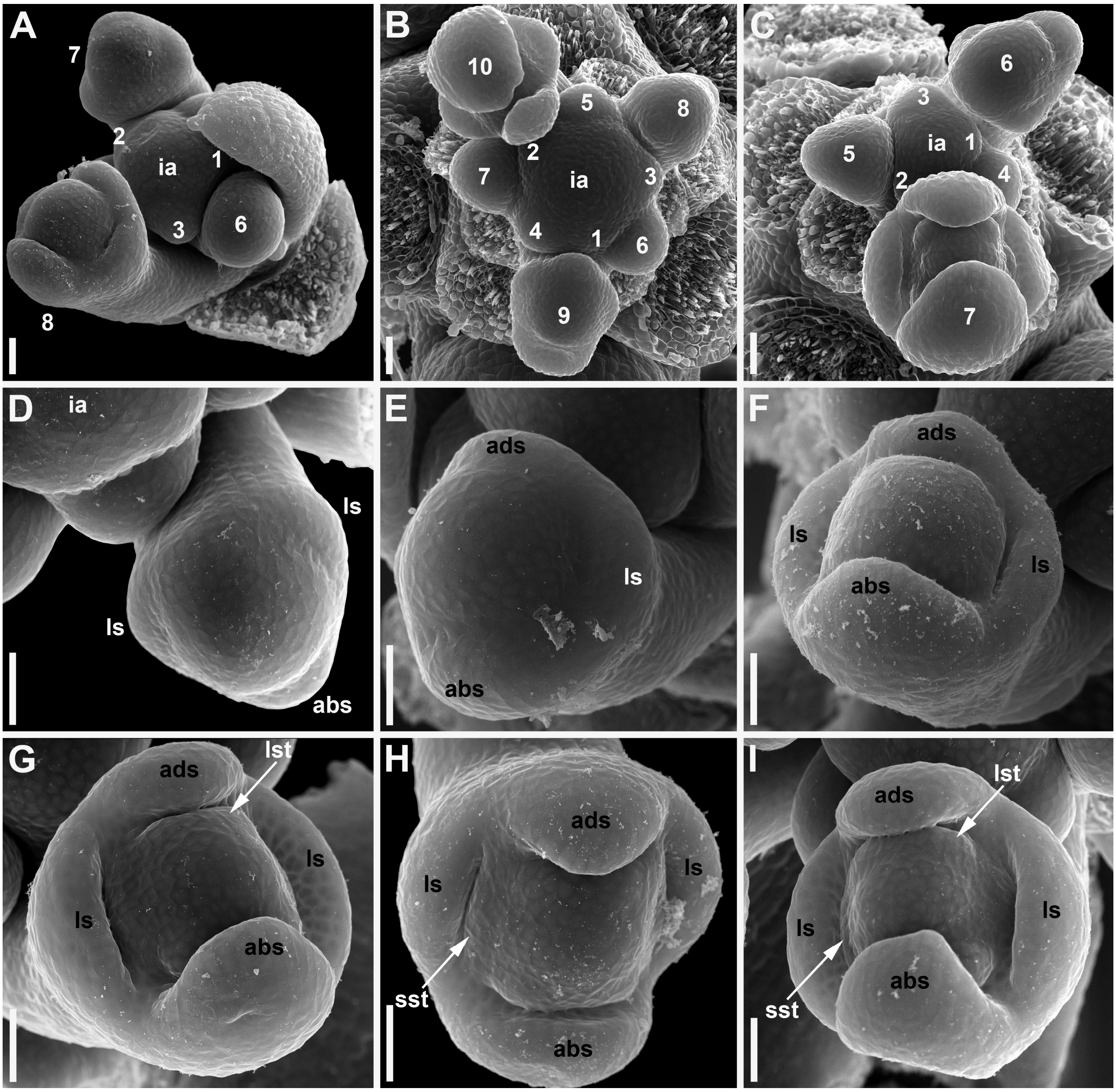

Figure S4. Early stages of flower development in *lel* plants of *C. bursa-pastoris* (SEM). (**A-C**). Inflorescence apices with flowers at sequential stages of early development. Flowers are numbered from the youngest to older ones. (**D-I**). Young flowers. (**D**). Flower with abaxial and lateral sepals initiated and adaxial sepal not yet clearly visible. (**E**). Side view of flower with all four sepals initiated. The abaxial sepal is larger than the adaxial one. All sepals are basally congenitally united. (**F**). Sepals are larger than in (**E**), but there is still no evidence of stamen initiation. The abaxial sepal is larger than the adaxial one, and the lateral sepals are smaller than the median ones. (**G-I**). The earliest evidence of androecium initiation as four bolsters situated between the four sepals and the nearly rectangular floral apex. The bolsters in front of the lateral sepals are less conspicuous and situated at a lower level than those in front of the median sepals. abs, abaxial sepal; ads, adaxial sepal; ia, inflorescence axis; ls, lateral sepal; lst, long stamens (i.e., those that will be long stamens in anthetic flowers); sst, short stamens (i.e, those that will be short stamens in anthetic flowers). Scale bars: 30 µm.

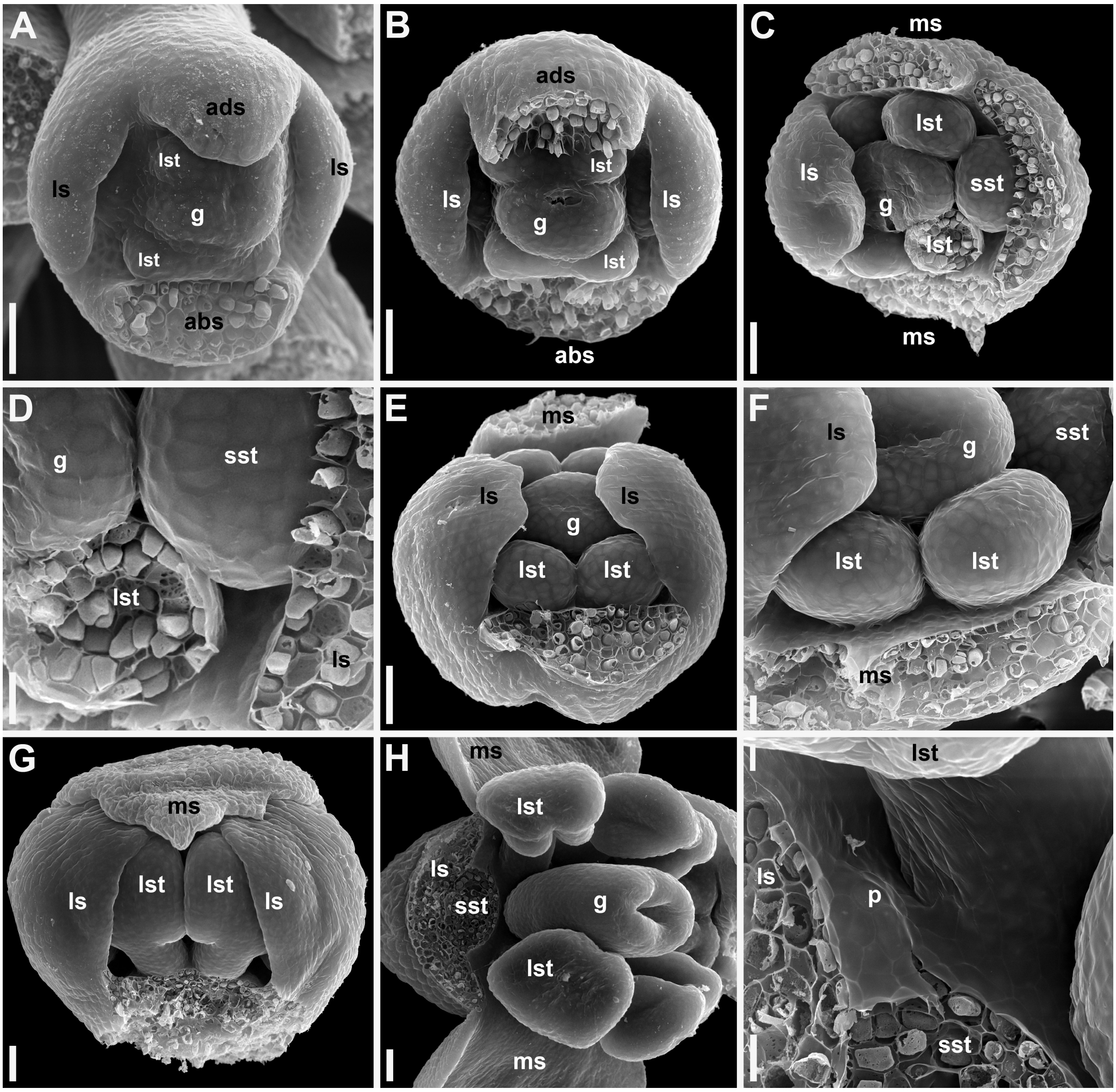

Figure S5. Androecium development and the earliest evidence of petal initiation in *lel* plants of *C. bursa-pastoris* (SEM). (**A**). Flower with the largest (abaxial) sepal removed. There is a bolster-shaped androecium primordium in front of the abaxial sepal; it will produce two long stamens whose positions can be already recognized on each side of the bolster. It is not clear whether the two adaxial long stamens initiate as a common primordium, but they are clearly delayed in development relative to the two abaxial long stamens. Primordia of the two short stamens are situated in front of lateral sepals; they are small and the right hand one is not well visible. (**B**). Stage slightly older than in (**A**). Both median sepals covered androecium and gynoecium and were removed during dissection. The primordia of the two short stamens are clearly visible in from of each of the lateral sepals. The two abaxial long stamens are clearly united, but the two adaxial long stamens seem to be free. (**C-F**). Stages with further differentiation of stamens and gynoecium but yet no traces of petal initiation. Connection between abaxial long stamens cannot be recognized anymore. A depression is present in the centre of gynoecium. (**D**) is an enlarged detail of (**C**). (**G-I**). Flowers with stamen filaments clearly visible and the first evidence of petal initiation. (**G**). Side view of flower with one median sepal removed. Note the gaps on either sides of the visible pair of long stamens. A petal primordium can be seen in the right hand gap. (**H**). Flower with a sepal and a stamen removed. (**I**) detail of (**H**) showing a petal primordium. abs, abaxial sepal; ads, adaxial sepal; g, gynoecium; ls, lateral sepal; lst, long stamens (i.e., those that will be long stamens in anthetic flowers); ms, median sepal (in cases where we cannot precisely distinguish between ads and abs); p, petal; sst, short stamens (i.e, those that will be short stamens in anthetic flowers). Scale bars: 30 µm (**A-C,E,G,H**), 10 µm (**D,F,I**).

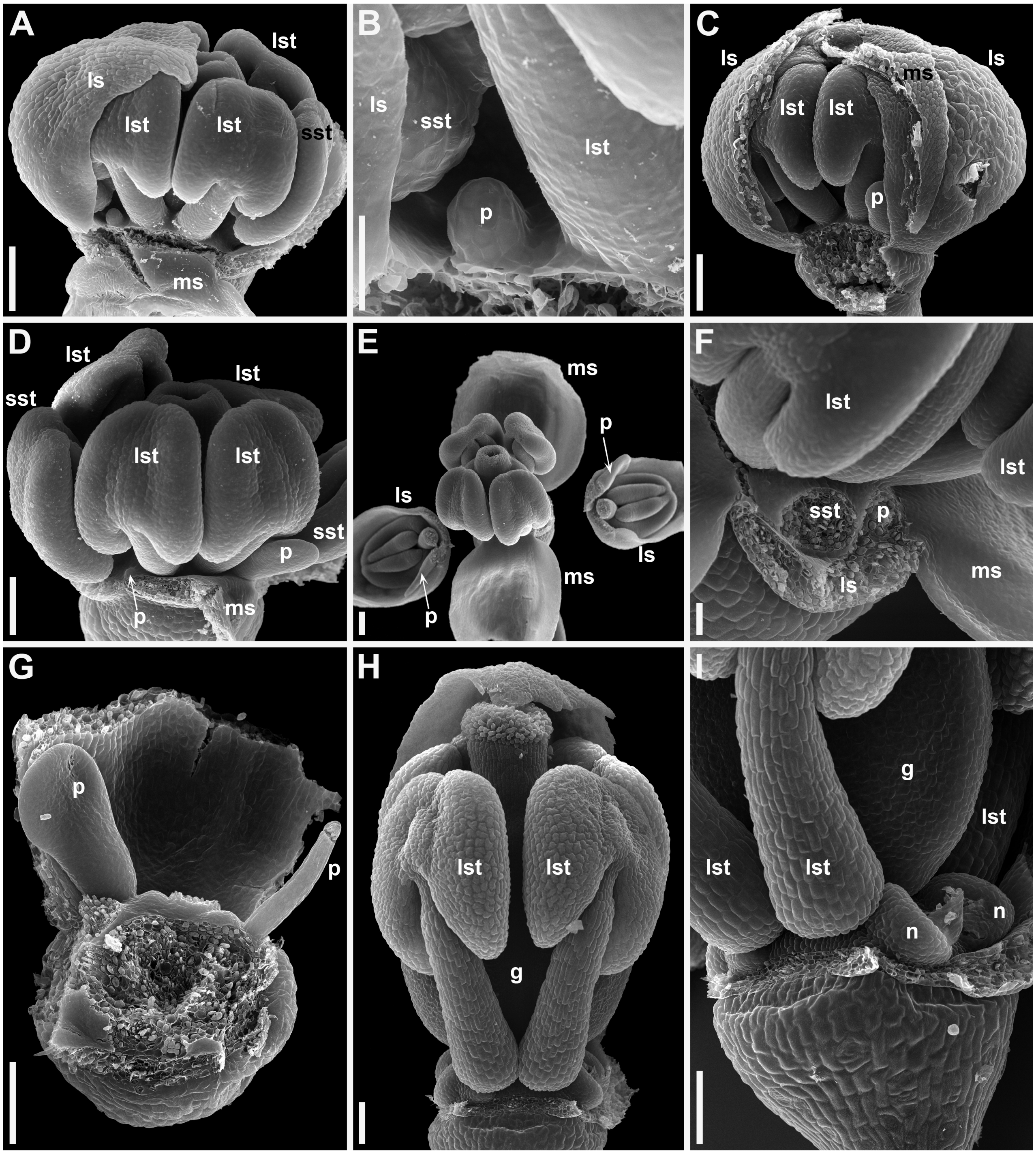

Figure S6. Late stages of flower development in in *lel* plants of *C. bursa-pastoris* (SEM). (**A**). Side view of flower with three of four sepals removed. A young petal is present left to the removed median sepal. There is no petal right to the median sepal (though there is enough space for its initiation). (**B**). Detail of (**A**) showing the petal. (**C**). Side view of flower with a median sepal partially removed. There is a petal right to the median sepal, but no petal left to it. (**D**). Side view of flower with sepals removed. One of the two visible petals is very small. (**E,F**). Dissected flower with four sepals, two petals, six stamens and two carpels. (**E**). Top view. (**F**). Close up of side view showing the absence of a petal left to the lateral sepal. G. Flower with all organs but petals removed. One of the two petals is filament-like. (**H,I**). Pre-anthetic flower with three sepals and both short stamens removed. Note the absence of petals. g, gynoecium; ls, lateral sepal; lst, long stamens (i.e., those that will be long stamens in anthetic flowers); ms, median sepal; n, nectary; p, petal; sst, short stamens (i.e, those that will be short stamens in anthetic flowers). Scale bars: 100 µm (A,C-E,G-I), 30 µm (B,F).
